# Schistosomiasis Consortium for Operational Research and Evaluation (SCORE): Its Foundations, Development, and Evolution

**DOI:** 10.4269/ajtmh.19-0785

**Published:** 2020-05-12

**Authors:** Daniel G. Colley, Julie A. Jacobson, Sue Binder

**Affiliations:** 1 Schistosomiasis Consortium for Operational Research and Evaluation, Center for Tropical and Emerging Global Diseases, University of Georgia, Athens, Georgia;; 2 Department of Microbiology, University of Georgia, Athens, Georgia;; 3 Bridges to Development, Seattle, Washington

## Abstract

The Schistosomiasis Consortium for Operational Research and Evaluation (SCORE) was established in late 2008 to conduct operational research that would inform practices related to the control and elimination of schistosomiasis. This article traces SCORE’s beginnings and underpinnings. These include an emphasis on openness and contributing to the development of a cohesive schistosomiasis control community, building linkages between researchers and national programs, and focusing on answering questions that will help Neglected Tropical Disease program managers to better control and eliminate schistosomiasis. It describes the development and implementation of SCORE’s multiple projects. SCORE began by drawing on advice from a broad range of experts by holding wide-ranging meetings that informed the priorities and protocols for SCORE research. SCORE’s major efforts included large, multicountry field studies comparing multiple strategies for mass drug administration with praziquantel, assessment of approaches to elimination, evaluation of a point-of-care assay for field mapping *Schistosoma mansoni*, and increasing the sensitivity of a laboratory-based diagnostic. SCORE also supported studies on morbidity due to schistosomiasis, quantification of vector snails and the detection of schistosome infections in snails, and changes in schistosome population genetics under praziquantel drug pressure. SCORE data and specimens are archived and will remain available for future research. Although much remains to be carried out, our hope is that through the already published articles and SCORE results described in this supplement, we will have provided a body of evidence to assist policy makers in the development of judicious guidelines for the control and elimination of schistosomiasis.

## INTRODUCTION

This article is being written on the 10-year anniversary of the establishment of the Schistosomiasis Consortium for Operational Research and Evaluation (SCORE) and as the SCORE project nears completion. It describes the context in which SCORE was developed and the conceptual basis on which it was founded. It also lays the groundwork for subsequent articles in this supplement to the *American Journal of Tropical Medicine and Hygiene*, which summarize many of the findings and lessons learned through SCORE studies. The final article in this supplement looks to the future—providing the thoughts of the SCORE secretariat—with input from the research consortium and its partners, regarding where SCORE will leave off and what will hopefully follow.^1^

## THE ORIGINS OF SCORE

### Development and publication of the Schistosomiasis Research Agenda.

In December 2007, Daniel Colley and Evan Secor published an article entitled “A Schistosomiasis Research Agenda.”^2^ This article sought to provide a comprehensive and cohesive summary of critical needs in schistosomiasis research. It drew from the agendas of existing programs, such as those at the WHO and the U.S. National Institute for Allergy and Infectious Diseases (NIAIDs) of the NIH, and input from people around the world. Ideas and comments were solicited from more than 350 people, and more than 150 people actually contributed.

The goals of the agenda development process and publication included increasing interactions among the schistosomiasis community, which until then, was perceived as highly fragmented, and stimulating interest of funders to address critical research needs. Colley and Secor hoped that the process for developing the agenda—which was highly interactive—and its publication would lead to more interdisciplinary networks focused on schistosomiasis research, standardization of protocols for multicenter studies, increased use of repositories, recruitment of trainees, enhanced mentoring of junior researchers, enlistment of outside experts into schistosomiasis work, and a higher profile for schistosomiasis research that would lead to development and adoption of evidence-based approaches to control by the global health community. In regard to increasing funding, the hope was that investigators and funding organizations would respond to a community-generated, comprehensive agenda by identifying particular areas in the agenda in which they could participate and provide support.

In September 2007, while the agenda manuscript was “in press,” Colley made an invited presentation about the schistosomiasis research agenda at a workshop at the NIAID. In his presentation, he suggested that different funders focus on different parts of the agenda. For example, NIAID should focus on various basic research aspects, whereas others should fund operational research. Julie Jacobson, senior program officer of the Infectious Diseases program at the Bill & Melinda Gates Foundation (BMGF), was in the audience.

Previously, the foundation had funded large field programs as independent investments in multiple neglected tropical diseases (NTD), including schistosomiasis (through the Schistosomiasis Control Initiative [SCI] at Imperial College), as exploratory one-time investments. Although this approach resulted in some very important early programmatic findings, the BMGF did not consider this the best way forward to address the interrelated needs of control programs, including to bolster much needed evidence-based guidance and share findings collaboratively among multiple, broadly based researchers. The collaborative approach taken by the Schistosomiasis Research Agenda^2^ showed the potential to focus the next investments to build on progress of previous investments in a more comprehensive way and, thus, to speed progress and build the needed evidence base for programs. The approach used by the BMGF on lymphatic filariasis (LF) seemed a good model to operationalize the research agenda, with multiple researchers aligned around shared, programmatically relevant goals. Colley’s work on the schistosomiasis research agenda, strong standing in what was thought of as a fragmented schistosome research community, and collaborative nature made him an excellent choice to apply a consortium approach to the operational research considered necessary to enhance control and move toward possible elimination of schistosomiasis.

On January 9, 2008, Jacobson called Colley at the University of Georgia (UGA), where Colley was the director of the Center for Tropical and Emerging Global Diseases, to discuss BMGF interest in having him lead a large operational research effort related to schistosomiasis, focused on *Schistosoma mansoni* and *Schistosoma haematobium*. The emphasis would be on research that was likely to have a major impact on schistosomiasis control and elimination in a relatively short time frame as programs were just starting to scale up. Research on vaccines and new pharmacologic treatments, which was thought unlikely to impact control programs in the short term and direct work on *Schistosoma japonicum*, was explicitly excluded from the effort Jacobson was proposing. Furthermore, this program would work with existing NTD control programs when possible and would make an effort to engage the broad schistosomiasis community.

Following that discussion, SCORE began to take shape. In mid-February, the office of the President of UGA awarded Colley a developmental grant to support continued engagement with the broad schistosomiasis community, further define the most critical questions for SCORE to address, and garner ideas about how to structure a research consortium that could implement the range of studies potentially to be funded by a BMGF grant. Six meetings and multiple discussions with a wide spectrum of leaders from the schistosomiasis research and control communities, including the WHO, led to a consensus that the field studies would be largely based in Africa and would be most likely carried out through North–South partnerships involving both researchers and NTD program managers.

### Funding of SCORE.

Colley submitted the SCORE Letter of Intent to the BMGF on April 11, 2008, and it was accepted on June 2. The final proposal was submitted on September 15, 2008 at about the same time as the 2008 global financial crisis was forcing many funders, including the BMGF, to re-examine the extent and priorities of their investments. Jacobson remained a staunch supporter of the SCORE proposal and argued hard for the BMGF to support its objectives, believing in the high potential for impact, increased program efficiency, and alignment with the BMGF commitment to equity. In late November, the BMGF funded SCORE through the UGA Research Foundation, Inc. with a 5-year, $18.7 million grant. This represented an enormous commitment to schistosomiasis operational research, funding work that had not been and was unlikely to be funded by others. The BMGF has continued its support for SCORE for more than 10 years, including a June 2013 funding supplement focused on research related to elimination of schistosomiasis and multiple no-cost extensions.

## SCHISTOSOMIASIS CONTROL IN 2009

In 2009, it was estimated, based on a systematic review that used data from 2003, that 779 million people were at risk for schistosomiasis and more than 200 million were infected.^3^ Although specific schistosomiasis control measures, such as environmental changes that reduce snail habitat and the general effects of improved sanitation, had markedly reduced or eliminated schistosomiasis in some places, population growth and development projects, such as irrigation systems and dams, appeared to be exacerbating the problem in others.^4,5^

The mainstay of schistosomiasis control programs, based on WHO guidelines from 2001^6^ was preventive chemotherapy through mass drug administration (MDA) of praziquantel (PZQ), largely through schools, with a focus on morbidity control as defined by WHO, that is, reduction in high-intensity infections. SCI, funded by the BMGF, had recently demonstrated in several countries that MDA for schistosomiasis could be rolled out on a countrywide basis, given sufficient funding, country commitment, and good management.^7^ Nevertheless, there was recognition that significant functional morbidities were impacting infected individuals who did not have high-intensity infections, and most people at risk were not receiving treatment.^8^ Utzinger et al.^9^ estimated that treating all 128 million African children thought to be at risk for schistosomiasis according to the WHO guidelines would require between 192 and 384 million PZQ tablets every year.

The WHO guidelines were written at a time when PZQ was scarce and very expensive, and disease burden was relatively high. Therefore, they focused on controlling morbidity due to chronic heavy infections leading to severe disease, such as portal hypertension, hepatosplenomegaly, and carcinoma of the bladder,^10,11^ and on treatment of schoolchildren and not generally adults.

Propitiously, beginning in 2009, Merck KGaA (Darmstadt, Germany) pledged to donate 200 million tablets annually of PZQ for 10 years through WHO for countries to implement national programs to control morbidity due to schistosomiasis, mainly in school-aged children in sub-Saharan Africa. Then in 2012, as part of the London Declaration, the company pledged to incrementally increase their donation to reach 250 million tablets per year by 2016 and to continue at that level until 2020 in support of the WHO NTD Roadmap goals. The confluence of the findings by SCI that nationwide MDA was possible and this major donation of PZQ by Merck KGaA provided the impetus and opportunity for operational research such as that being proposed for SCORE into what might be the best MDA strategies, as well as other approaches and tools that could help decrease transmission and measure impact of interventions.

At the time the SCORE studies were being developed, the prevailing concept was that control of NTDs would be best carried out through integrated programs,^12^ which were expected to be more cost-efficient than stand-alone programs. With the support of Jacobson, it was decided that research within SCORE would focus on control and elimination of only schistosomiasis, although testing for soil-transmitted helminths (STH) would be included in studies of *S. mansoni* where it would not disrupt the schistosome-focused study design, and MDA for STH would be included where appropriate. The thinking behind this was that schistosomiasis poses several challenges for integration, and first determining how best to reduce schistosomiasis on its own would provide a better understanding of how to integrate schistosomiasis control into broader programs. Also, it was decided that study sites would be the areas with either *S. mansoni* or *S. haematobium*, but not mixed infections*.*

## SCORE STRUCTURE AND INITIAL RESEARCH PLANNING

### The SCORE Secretariat and Advisory Committee.

SCORE was managed by a very small Secretariat, which included individuals with a range of experiences and skills. At its maximum, the Secretariat included seven people, only two of whom were full-time.

An Advisory Committee to the SCORE secretariat was established, consisting of Paul Hagan, Stephanie James, Stephen McGarvey, Eric Ottesen, and Joseph Cook. First, Lorenzo Savioli, and then Dirk Engels and subsequently Gautam Biswas, the directors of the WHO/NTD program, graciously committed to always having someone in the WHO/NTD group serve as an ad hoc member of the SCORE Advisory Committee, thus ensuring a strong connection between SCORE’s studies and activities and the WHO/NTD team. First Lester Chitsulo and then Amadou Garba served in this capacity.

### Expert panels.

Very quickly after being funded, during 2009, SCORE held a series of seven expert panels to identify and explore research priorities for what were considered the most critical questions on the control and elimination of schistosomiasis. The topics for meetings, dates they were held, and numbers of attendees are listed in Table 1. Many of these panels involved not only those working on schistosomiasis but also experts on other NTDs or in other fields entirely.

**Table 1:** Schistosomiasis Consortium for Operational Research and Evaluation expert panels held in 2009 and number of attendees at each panel

Topic	Date	Number of attendees
1. Schistosome population structure under MDA	April 16–17	11
2. Existing data and experiences on control of schistosomiasis and other neglected tropical diseases when relevant	April 22–24	17
3. Monitoring of schistosome infections in snails	May 7–8	8
4. Gaining and sustaining control of schistosomiasis	June 3–5	10
5. Elimination of schistosomiasis	July 6–7	12
6. Monitoring morbidity changes under MDA	July 9–10	11
7. Development of a true diagnostic test for human schistosomiasis	September 30–October 1	15

MDA = mass drug administration.

These seven meetings (and a subsequent one on snail control in 2013) were essential for the development of the protocols that were the basis of the multiple studies pursued by SCORE. The underlying premise on which all the protocols were eventually based was whether the answers from the research would be likely to help a program manager do his or her job better. Meetings were highly interactive, with excellent participation by all. Discussions centered around scientific/programmatic issues, but by necessity also included political and security issues as well as costs and other practicalities.^13^ There were often strong disagreements, but these grew out of a shared desire for a deeper understanding of the issues and a commitment to making the SCORE research as good and meaningful as possible.

### Protocol development and process for funding.

The expert panel meetings resulted in outlines of priority studies. These were subsequently refined by SCORE secretariat into draft requests for applications (RFAs), most of which were reviewed by the SCORE Advisory Committee and other colleagues before being finalized. The final RFAs were sent to investigators with track records in similar operational research. For the large studies on gaining and sustaining control, a critical requirement was the buy-in of the national NTD programs of the countries where the proposed studies would be conducted.

Multiple applications were received. These were evaluated by the Secretariat with assistance from the SCORE Advisory Committee and funding decisions made. From 2009 to 2018, a total of 48 sub-awards, some with multiple activities added during the years, have been made through SCORE.

## THE SCORE RESEARCH PORTFOLIO

As noted earlier, the guiding principle by which studies were considered and designed was based on the simple question: “What strategies and tools will help NTD program managers do their job better?” Because in 2009, the mainstay tool was MDA with PZQ, the SCORE research portfolio initially focused on large field studies of alternative approaches to MDA and field evaluation of a mapping tool for *S. mansoni*, with other research quickly following. The portfolio evolved, both as a result of early findings from SCORE and other studies and from new priorities from the BMGF.

### Alternative approaches to MDA.

An attempt by SCORE to conduct a meta-analysis of studies on control of schistosomiasis found a scarcity of randomized trials and other quality studies that compared approaches with achieving reductions in schistosomiasis prevalence and intensity. This reinforced the need for better data on the impact by alternative approaches. Thus, a high priority for SCORE was research on how best to use what were, at the time, limited supplies of PZQ—whether through community-wide treatment (CWT) or school-based treatment (SBT) and whether to treat more villages by doing MDA every other year, with the year off being referred to as “drug holiday.” Such data were needed for planned revisions of the WHO guidelines related to schistosomiasis control, and to provide an evidence base for new thresholds for action and recommended interventions.

An issue raised in SCORE’s expert panel meetings was whether it was ethical to use approaches to MDA that were inconsistent with the existing WHO guidelines. Chitsulo, the WHO ad hoc member of SCORE’s Advisory Committee at the time, noted that the guidelines were based on little hard evidence and needed a scientific basis. Given that the benefits of annual MDA versus every-other-year had not been established, and that it was unclear if a program would provide more benefit by treating twice as many communities every other year versus a smaller number annually, the inclusion of holidays was thought by the participants to be ethical. Chitsulo said, “You cannot evaluate the guidelines if you go by the guidelines.”

In addition, it was noted that all studies would be reviewed by human subjects review boards, including boards in-country. Also, after the final data collection, all villages were to be receiving MDA; so at the end of the study, each participating village would have had at least two consecutive rounds of MDA.

Other issues discussed included the prevalence cutoffs to be used, treatment of adults in villages receiving CWT and the frequency of the MDA.

The goal of developing data that would evaluate current guidelines and contribute to new, evidence-based guidelines led to SCORE’s largest field studies, referred to as the SCORE “gaining and sustaining control studies.”^14^ These studies were carried out in five African countries: Côte d’Ivoire, Kenya, Mozambique, Niger, and Tanzania. The protocols for these studies involve various combinations of community-wide MDA, school-based MDA, and drug holidays over a period of 4 years, followed by final data collection in the fifth year. The primary outcome of interest was change in prevalence and intensity of schistosomiasis among children ages 9–12 years. Data were also collected at given times on incoming first-year students (ages 5–8 years) and adults from 20 to 50 years of age. In addition, data were collected on factors that might prove useful in predicting a community’s response to MDA, such as village-level data about sanitation, occupations, population shifts, snail infections and abundance, and changes in schistosome population structure under drug pressure. Other articles in this *Journal* supplement address the results of the large field studies on gaining and sustaining control of schistosomiasis.^15–17^

One challenge that SCORE overcame with the help of partners was related to the supply of PZQ for these large field studies. Praziquantel was needed for 4–5 rounds of MDA (including MDA after final parasitologic testing) in 825 villages in the five countries. Because WHO/NTD initially promised to supply the PZQ needed for these studies, the SCORE proposal to the BMGF did not request funds for PZQ. Unfortunately, the WHO/NTD program could not deliver on their promise. Graciously, programs funded through SCI, the United States Agency for International Development, and the Department for International Development of the United Kingdom came to the rescue in the different countries. Although it took considerable discussion, there were times when SCORE was able to convince one NTD program manager in one country to provide expiring PZQ to another program manager in another country that needed PZQ for their SCORE study. One positive result of this change in the sources of PZQ in some countries was stronger links between the research programs and the national control programs that were managing the PZQ donations—an important aspect of these studies.

### Subtle morbidity.

The expert panel meeting on subtle morbidity reflected the state of the field. This included recognition that infected individuals without high-intensity infections could also experience significant morbidity, and that the commonly used parameters to measure subtle morbidity in schistosomiasis were not reliably indicative of the attributable fraction of morbidity actually due to schistosomiasis. Nevertheless, measurements were selected for study in cohorts of children entering the gaining control studies in Mozambique, Niger, Kenya, and Tanzania. Comparisons would be between children in villages in the arms with what was expected to be the most intensive MDA (annual CWT for 4 years) versus villages in the arms that would receive substantially less intervention (SBT biennially). The potential morbidity markers to be measured included anthropometric measures, abdominal or urogenital ultrasounds, measures of fitness and/or volitional activity, anemia, and quality of life.^18^

### Rapid answer projects (RAPs).

During the meetings of expert panels, participants described many questions they were being asked by program managers that likely had answers in the existing literature. In 2009, SCORE initiated the RAPs. Questions included whether adults could become reinfected with *S. haematobium* after treatment and how much benefit was added by two closely spaced PZQ MDAs versus a single MDA. Some of the RAPs that were developed provided findings to be tested in the larger SCORE field studies. This approach of synthesizing the existing literature on a focal topic pertinent to control has led to seven completed RAPs.^19^

### Elimination of schistosomiasis.

The purpose of the SCORE elimination studies was to conduct research on what integrated strategies might be able to stop transmission and achieve elimination.^20^ The first challenge in this effort was where in sub-Saharan Africa an elimination study could be conducted. After an extensive process, the archipelago of Zanzibar was selected as the study site. Many factors contributed to this selection, including clear geographic boundaries; strongly stated political support, including from the President of Zanzibar; commitment to biannual MDA by the Ministry of Health and partners; and other resources available on the islands.

The decision was difficult. Based on his early experience in the 1970s with the Research and Control program on St. Lucia,^21^ Colley had concerns about whether the broader community would accept the results of research on schistosomiasis elimination on islands as having general applicability. The late Likezo Mubila of WHO/Regional Office for Africa convinced Colley otherwise, providing persuasive insights and fully supporting SCORE’s investment in Zanzibar. Similar concerns were raised at the BMGF. However, a consensus was reached and plans for research on elimination on Unguja and Pemba, the main islands of Zanzibar, moved forward. Subsequently, as SCORE and partners, such as the Ministry of Health, SCI, the Natural History Museum (NHM), and the Swiss Tropical Public Health Institute were planning the research studies; a collaboration of a wide range of multiple agencies and investigators was created, which called itself Zanzibar Elimination of Schistosomiasis Transmission.^22,23^ The SCORE Zanzibar Elimination Study became the research component of this collaborative effort.

In May 2012, the World Health Assembly (WHA) issued WHA Resolution 65.21 that called for the expansion of schistosomiasis control programs and to “initiate elimination campaigns, where appropriate.”^24^ In keeping with this resolution and the adoption of the WHO NTD Roadmap goals in the London Declaration, in June 2013, the BMGF provided SCORE with an additional $3,468,375 and an extension of the project to December 31, 2017 to conduct supplemental work on elimination and other projects evolving from SCORE findings.

The objectives of this supplement were to 1) evaluate approaches to elimination of *S. mansoni* transmission, 2) evaluate approaches to elimination of *S. haematobium* in areas with seasonal transmission, 3) conduct operational research on innovative approaches to snail control, 4) conduct additional RAPs to synthesize the existing literature to provide guidance for programs about use of niclosamide and about use of sanitation measures, and 5) conduct meetings to develop sampling schemes and tools to assess progress towards and achievement of elimination.

One objective of the supplement was to evaluate approaches to elimination of *S. mansoni* transmission. Unfortunately, after extensive mapping, the planned *S. mansoni* intervention studies were ultimately not completed in either Rwanda because of lack of national government buy-in or Burundi because of civil unrest. However, the extensive mapping by Kato–Katz, the point-of-care circulating cathodic antigen (POC-CCA) assay, and, in a subset of specimens, the up-converting phosphor lateral-flow circulating anodic antigen (UCP-LF CAA) (see below) provided critical information about performance of both Kato–Katz testing and POC-CCA in an area of low prevalence and reinforced the message that there was more schistosomiasis in many “low-prevalence” areas than had previously been believed.^25,26^

The supplement also led to a major field study, the Seasonal Elimination Study on *S. haematobium,* which is currently being completed in Côte d’Ivoire and incorporates both MDAs and snail control, with timing of these two interventions based on the seasonality of transmission.^27,28^ In addition, in regard to snail control, SCORE held a meeting focused on various potential approaches as well as mollusciciding. SCORE also then pursued several studies on predatory crustaceans.^29^

### Development of mapping and diagnostic tools needed for control and elimination.

It was clear that to control schistosomiasis to moderate or low levels of prevalence and intensity of infection more sensitive mapping tools were needed, as well as a test that would be both highly sensitive and highly specific. The previously mentioned 2009 meeting on diagnostics included both individuals working with parasite diagnostics and those using cutting-edge technologies for other purposes (e.g., sniffing for low concentrations of nerve gases and automated PCR for biological agents). The discussions included tools for mapping, but largely focused on possible diagnostics with very high sensitivity and specificity.

Many laboratories that had developed “boutique assays” for research mapping and diagnostics that they used in their own studies or perhaps were used by one or two other groups in collaboration hoped that SCORE would be able to provide support. Unfortunately, SCORE was not funded for product development and, therefore, did not have the funding, the technical staff, or time needed to develop and evaluate new mapping tests that were not ready or near-ready for field work.

Shortly before SCORE was funded, the POC-CCA urine assay for *S. mansoni* was commercialized and made available for purchase (Rapid Medical Diagnostics, Pretoria, South Africa). This assay uses monoclonal antibodies to detect a glycan (circulating cathodic antigen) vomited by adult worms into the blood stream, cleared in the kidneys, and detectable in urine.^30^ The assay is not only more sensitive than the Kato–Katz assay for *S. mansoni* at low prevalence but it also obviates the need for collecting stools and for trained microscopists. Although it appeared to perform well in laboratory and small-scale field settings, it had not been extensively evaluated in the field in endemic areas with different levels of prevalence and intensity of infections. The SCORE Five-Country Study, which compared the POC-CCA versus the Kato–Katz assay, and several subsequent investigations are summarized in the article on the POC-CCA.^31^

At the time, it was already recognized that the POC-CCA was not useful for mapping for *S. haematobium*^32^; however, there was no new field assay for *S. haematobium* near ready for field-testing. SCORE’s portfolio did not include product development; therefore, a mapping tool to replace the urine dipstick for hemoglobin or microscopy-based urine filtration for *S. haematobium* eggs was not pursued. Later, limited resources were used to try to refine and test *S. haematobium*–mapping tools, but these attempts did not work well enough to warrant further investment of project funds.

Regarding the highly sensitive and specific diagnostic, discussions at the 2009 meeting covered a range of approaches, including nucleic acid, antigen, and antibody tests. It was decided that SCORE would support the further development of the laboratory-based UCP-LF CAA assay, housed at the Leiden University School of Medicine. This sensitive assay detects a different glycan antigen than that detected by the POC-CCA but is also produced by both *S. mansoni* and *S. haematobium* adult worms, the CAA.^33^ The specific goals of SCORE support were to make the UCP-LF CAA assay as sensitive as possible, in hopes of being able to detect single-worm infections, and to evaluate its potential as a confirmatory assay for proof of cure or lack of infection. The envisioned uses of the assay included assessing the results of field tools such as the POC-CCA, accurately measuring prevalence and intensity as places approach elimination, and determining whether PZQ treatment was curative in an individual. Results of the SCORE investment in UCP-LF CAA development and findings from its use in SCORE studies are summarized later in this supplement.^34^

### Schistosome detection in snails.

In addition to diagnostics for humans, diagnostics for snails could be critical for evaluating elimination efforts and assessing force of transmission in studies of gaining and sustaining control. Although initially SCORE planned to invest in snail diagnostic test development, the SCORE meeting of experts concluded that existing methods were adequate for SCORE purposes. Furthermore, it was thought that other research funding, for example, for development and validation of loop-mediated isothermal amplification techniques would likely provide useful tools for such xenomonitoring by the time they would be needed for future elimination programs. Rather than focusing on development of more or better tools for snail infection detection, SCORE layered snail collection studies that included measurement of patent infections within some of the gaining control studies^35^ and the Zanzibar Elimination Study, and eventually the Seasonal Elimination Study in Côte d’Ivoire.^28,29^

### Schistosome population genetics.

While the need for research on most of the topics selected by SCORE was somewhat self-evident, there was considerable early discussion about the inclusion of schistosome population genetics, primarily about whether such work was too much in the realm of “basic science” for SCORE. The ultimate decision to support schistosome population genetics research was based on the concern that if drug resistance could not be detected until it could be measured clinically, it would be too late to ensure the continued utility for PZQ–the only drug currently being used to treat schistosomiasis. Therefore, schistosome population genetics studies were designed to provide insights into potential changes in schistosome population structures under varying levels of MDA pressure. Should changes occur, the hope was that measurement of these changes could be developed into an early warning system for potential PZQ resistance.

Because the genome of *S. mansoni* was published, adequate information about microsatellites (the tool at the start of these studies) was available to characterize *S. mansoni*. However, there was much less experience with *S. haematobium* microsatellites. With SCORE support, a consortium involving UGA, the NHM (London, United Kingdom), and Centro de Pesquisas “Rene Rachou”/FIOCRUZ (Belo Horizonte, Brazil) conducted low-coverage genomic sequencing to identify thousands of microsatellite loci from a recent field isolate of *S. haematobium* from Zanzibar.^36^

As part of some of SCORE’s gaining control studies and the Zanzibar elimination study, well-characterized cercarial and miracidial specimens were collected and banked (rather than being analyzed immediately) in anticipation that over time costs would fall and better gene sequencing methodology would be developed. Genetic analyses were subsequently initiated. The information yielded thus far has contributed in several different and somewhat unexpected ways that are summarized in the article by Webster et al.^37^ Also, the collected specimens will provide rich material for analysis for years to come.

The inclusion of schistosome genomic studies led to another contribution by SCORE. The banking of specimens described above and of snail specimens from SCORE and other studies was possible because of the funding by the Wellcome Trust of a joint proposal from the NHM and SCORE. The Schistosomiasis Collection at the NHM (SCAN)^38^ is housed at the NHM. Adrian Emery is the principal investigator and curator of SCAN. In addition to the vast number of specimens of both schistosome DNA and snails from the SCORE studies, SCAN has now obtained many specimens from other projects and provides an in-and-out repository service for investigators in the schistosomiasis community.

## CHANGES THAT OCCURRED OVER SCORE’S 10 YEARS

Several changes previously mentioned impacted the course and priorities of SCORE. For example, as mentioned, the increase in interest in the schistosomiasis community in elimination led to the supplemental funding and projects aforementioned and in another publication in this supplement.^27^

### Changes from outside of SCORE.

Some issues of great concern at the time SCORE was planned are less so now. The push for integration of NTD control programs became more refined as the NTD community learned more about what elements were best integrated. Programmatic drug distribution in the case of schistosomiasis became linked to STH programmatic distributions through schools, but it is less often combined with other programs because of differences in delivery platforms and targeted age groups, the often focal nature of schistosomiasis, and the inclusion of drug holidays based on levels of prevalence.

Technologic developments also impacted SCORE. The banking of schistosome specimens for later genomic testing was described previously. The major gaining and sustaining control studies were initiated as phone-based data collection was beginning to take hold, replacing paper-based data and use of personal digital assistants. During this time of transition in data collection, the five countries involved in gaining and sustaining control studies used three different approaches to data collection: paper-based, a phone-based system developed by the EpiCollect group at Imperial College, and a phone-based system developed using the Open Data Kit. The challenges associated with this are described in the supplement article on recommendations.^39^

### Standardization of data reporting and analyses.

It also became clear about midway through SCORE that a bigger effort was needed to standardize the way SCORE field studies reported data to the Secretariat and the way the large field studies analyzed data. This led to the development of the SCORE Uniform Data System and Statistical Analysis Plans.

### Cost assessment.

A high priority for SCORE was assessing not just the effectiveness of the interventions related to gaining and sustaining control but also the costs of their implementation. With the help of economists experienced in conducting NTD cost-effectiveness evaluations, SCORE included a cost assessment in the second or third year of each of the studies of gaining and sustaining control. Some of the difficulties in conducting these were recognized in advance, such as the difficulty of distinguishing between program and research costs. For example, SCORE studies required collection of three stool specimens among 9–12-year-old children in the *S. mansoni* studies, instead of the single stool that would be collected in a typical program. When research team vehicles were used to deliver PZQ, costs also were higher than those from routine programs.

Unfortunately, the instructions for data collection were not uniformly followed, some of the data could not be analyzed, and other results could not be explained except by assuming the data were faulty. In Kenya’s gaining control study,^40^ where on-site training was provided, the data quality was good, but the costs were deemed not relevant for program purposes because of the use of the high-cost research infrastructure from CDC’s presence at the Kenya Medical Research Institute.

### Consequences of SCORE studies on POC-CCA.

Major changes in thinking have resulted from the data being generated by SCORE, for example, related to the POC-CCA urine assay for *S. mansoni*. The Five-Country Study about the POC-CCA and subsequent studies by SCORE^41–43^ and others^44^ clearly showed that the Kato–Katz assay underestimated the number of people infected with *S. mansoni*, especially in areas with less than 50% prevalence by the Kato–Katz assay.

Although many published articles had previously shown that the Kato–Katz assay, while highly specific, has quite low sensitivity in areas with low or moderate prevalence and intensity of infection, the finding of so much more schistosomiasis than expected raised multiple issues related to how to control and eliminate schistosomiasis. SCORE redirected funds to answer some of the critical questions, for example, whether people who are trace-positive by POC-CCA in areas of very low prevalence contribute to transmission.^31,45,46^

### Persistent hotspots (PHS).

Another important SCORE finding is that there is a great deal more variability in village-level responses to MDA than expected. Whether MDA is given using CWT or SBT, and whether given biannually, annually, or biennially, prevalence and intensity decline nicely in some villages, but not in others. Some villages may even have increased prevalence and, to a lesser degree, intensity, in the face of multiple MDAs. The SCORE program has termed these villages with poor response to multiple rounds of MDA as “persistent hotspots” (PHS). Additional SCORE evaluations of this issue are discussed in another article in this supplement^17^ and have been detailed previously.^47–49^

In October 2015, SCORE held an ad hoc meeting, scheduled between the 2015 Coalition for Operational Research on Neglected Tropical Diseases meeting and the American Society for Tropical Medicine and Hygiene meeting in Philadelphia, to discuss potential next steps in defining and understanding this variability in communities to MDA. The meeting was attended either in person or by Skype by 12 SCORE investigators and included a WHO representative. The discussion was wide-ranging, including what to call these areas of concern, how they should be defined, what was causing them, and what research might be conducted to better understand them. Once SCORE’s data exposed the existence of PHS in the face of well-conducted MDA studies and the subsequent publication of articles on this topic,^47–49^ others also focused on their occurrence in other large studies. This has led to an appreciation that PHS are real and need to be addressed in some way if programs are ever going to truly control or eventually eliminate schistosomiasis. The question of early identification of PHS and what to do when they are identified is now playing a role in WHO’s efforts to develop new guidelines for the control and elimination of schistosomiasis.

### Snail control.

At one time, snail control was the major means of schistosomiasis control. In several places, it had been demonstrated that it could be successful if consistently applied.^50^ Nevertheless, by the time that SCORE began, consideration of snail control as a means of controlling schistosomiasis had gone out of practice. However, as elimination appeared to be within reach and has become a larger focus, all tools available to suppress transmission and stop infection needed to be considered as part of the armamentarium, and snail control became an essential part of SCORE’s Zanzibar and Seasonal Elimination Studies.

Because the best snail control methods and approaches to monitoring were unclear, SCORE held a meeting in 2013 to discuss potential ways to control snails, including environmental, biologic, and chemical. Among the possibilities discussed were use of various snail predators or competitors, physical or herbicide-mediated habitat modifications, and molluscicides. Although many promising ideas were generated, none except the use of niclosamide was field ready, so all SCORE studies involving snail control used this chemical. A side benefit has been the training of individuals in Zanzibar and Côte d’Ivoire on how to apply niclosamide and monitor impact.

In addition, SCORE published an RAP on snail control,^51^ which synthesized the literature, again showing that when done properly, snail control impacts transmission. Some of the findings and contributions of snail control in the SCORE studies are provided in another article in this supplement.^29^ Modeling work by SCORE also emphasized the value of snail control in elimination efforts.^52^

Both in relation to the increased emphasis on elimination of schistosomiasis, and potentially in relation to address PHS, WHO has recently initiated a renewed effort related to snail control and has published an updated manual on snail control and held two workshops on it in sub-Saharan Africa.^53^

### Creating a community and ensuring SCORE findings have impact.

A broad interchange and advocacy role was embedded in SCORE’s third objective: to provide evidence-based findings to assist in the development of new guidelines and assistance to NTD program managers. As described in the section about the creation of SCORE, a critical aspect of SCORE was the fostering of a community committed to control and elimination of schistosomiasis, integration of research and programs at the country level, and extensive involvement with WHO.

The SCORE annual meetings, held at UGA every year starting in 2011, provided a forum for very frank and open discussions among the Secretariat, the principal investigators of the multiple sub-awards or their designees, program managers, representatives from the BMGF, WHO, and the Advisory Committee, along with occasional others from outside SCORE, about their progress or the lack thereof, and challenges as well as successes.

Secretariat members or representatives from among the funded projects also attended all the COR-NTD annual meetings, multiple NTD NGO Network (NNN) meetings, AFRO’s NTD program managers’ meetings, and a variety of meetings at the WHO/NTD headquarters. Of critical importance was the SCORE team reporting into the WHO NTD Strategic and Technical Advisory Group (STAG) Monitoring and Evaluations working group, where data were discussed and if appropriate, passed to the STAG for adoption into WHO recommendations and programs. These meetings provided an opportunity to share SCORE’s findings with a much wider audience of those involved in NTD control and elimination and to get input from those working similarly on other NTD diseases. SCORE was also fortunate to be granted a symposium in each of the last 10 American Society of Tropical Medicine and Hygiene annual meetings, which provided a broader stage for discussing SCORE’s projects, challenges, and findings and getting input on important issues such as PHS. To help ensure that the findings from SCORE research are quickly adopted by organizations that are funding or implementing schistosomiasis control efforts, SCORE has involved WHO at every step and has involved itself with a variety of expert committees.

### Changes in perspectives at the BMGF.

During the course of SCORE, several changes occurred at the BMGF that affected direction and implementation, including the increased emphasis on elimination and the standardizing analyses in the gaining and sustaining and elimination studies.

## CHALLENGES AND OPPORTUNITIES BEYOND SCORE

Much has been learned from the SCORE studies and other work conducted during the years of SCORE. For example, SCORE studies reinforced the notion that MDA is a critical tool for decreasing prevalence of high-intensity infections and reducing morbidity. However, SCORE studies also make it clear that new approaches will be needed. It is clear that some of the contributions from SCORE in terms of PHS, snail control, and more sensitive assays will be critical to the way forward. The SCORE datasets, schistosome genetic sequences, and specimens in SCAN should be useful for years to come.^1^

The end of SCORE is coinciding with the formulation of new WHO guidelines on schistosomiasis, and now both control and elimination are on the docket. One of the key insights from SCORE has been that, although the strategy for control of schistosomiasis was predicated on the strategy for the control of lymphatic filariasis (LF), schistosomiasis is not LF. Lymphatic filariasis control and elimination is based on annual MDA for 5 or 6 years to kill microfilariae (the infectious stage), and it then takes up to a year before their production resumes. Therefore, treatment stops continued transmission for up to a year. With schistosomiasis, someone can be infected immediately after treatment if they go into water inhabited by infected snails, and they can infect snails within 1–2 months of infection. Thus, the risk of infection and of infected people contributing to transmission is cumulative between annual MDAs. The SCORE data on PHS and trajectories of village responses to MDA emphasize the need for new approaches to monitoring and control in PHS and will be critical in formulating new guidelines. Another article in this supplement will discuss, in more detail, the thoughts of the SCORE secretariat and sub-awardees on the way forward beyond SCORE.^1^

Clearly, there are other critical issues related to schistosomiasis that were not addressed by SCORE, such as female genital schistosomiasis, how to treat those younger than 5 years, and what mapping/monitoring/impact assessment strategies will be both effective in determining PHS and realistic in terms of what an NTD program manager can actually do. Is it possible to confirm elimination and how do you detect early recrudescence? Will there be an effective vaccine by the time it is needed?^54^ Will drug resistance develop, putting us back into the 1970s with no role for preventive chemotherapy?^55^ These are issues that will need to be and will be dealt with beyond SCORE. With the support of the BMGF, many other organizations and agencies, and many other individuals, SCORE has had a good run and has, we believe, produced positive findings that are already helping shape the future of schistosomiasis control and elimination.
